# Transcatheter tricuspid valve replacement using the LuX‐Valve Plus system: 1‐year compassionate use outcomes

**DOI:** 10.1002/ejhf.70010

**Published:** 2025-08-18

**Authors:** Lukas Stolz, Neil Fam, Geraldine Ong, Pedro Villablanca, Ahmad Jabri, Ole de Backer, Jacob Eifer Mølller, Didier Tchétché, Omar Oliva, Kent Chak‐Yu So, Yat‐Yin Lam, Azeem Latib, Edwin C. Ho, Andrea Scotti, Augustin Coisne, Arnaud Sudre, Julien Dreyfus, Mohammed Nejjari, Ignacio Cruz‐Gonzalez, Rodrigo Estévez‐Loureiro, Manuel Barreiro‐Perez, Guillaume Leurent, Erwan Donal, Guillaume Bonnet, Thomas Modine, Raj Makkar, Dhairya Patel, Karl‐Patrik Kresoja, Philipp Lurz, Jörg Hausleiter

**Affiliations:** ^1^ Medizinische Klinik und Poliklinik I, LMU Klinikum Munich Germany; ^2^ German Center for Cardiovascular Research (DZHK), partner site Munich Heart Alliance Munich Germany; ^3^ St. Michael's Hospital University of Toronto Toronto ON Canada; ^4^ Division of Cardiology, Department of Structural Heart Disease Henry Ford Health System Detroit MI USA; ^5^ The Heart Center, Rigshospitalet Copenhagen Denmark; ^6^ Clinique Pasteur Toulouse France; ^7^ Division of Cardiology, Department of Medicine and Therapeutics, Prince of Wales Hospital Chinese University of Hong Kong Hong Kong SAR China; ^8^ Montefiore‐Einstein Center for Heart and Vascular Care, Montefiore Medical Center Albert Einstein College of Medicine Bronx NY USA; ^9^ University of Lille, Inserm U1011‐EGID, Centre Hospitalier Universitaire Lille, Institut Pasteur de Lille Lille France; ^10^ Cardiology Department Centre Cardiologique du Nord Saint‐Denis France; ^11^ Department of Cardiology Complejo Asistencial Universitario de Salamanca Salamanca Spain; ^12^ CIBER‐CV, Biomedical Research Institute of Salamanca (IBSAL) Salamanca Spain; ^13^ Department of Interventional Cardiology Hospital Alvaro CunqueiroInstituto de Investigación Galicia Sur, Servizo Galego de Saude Vigo Spain; ^14^ Instituto de Investigación Galicia Sur, Servizo Galego de Saude Vigo Spain; ^15^ Department of Cardiology, Centre Hospitalier Universitaire Rennes, Inserm, LTSI‐UMR 1099 University of Rennes 1 Rennes France; ^16^ Department of Cardiology and Cardiovascular Surgery, Heart Valve Center, Institut Cœur Poumon CHU de Bordeaux Bordeaux France; ^17^ Department of Cardiology Smidt Heart Institute, Cedars‐Sinai Medical Center Los Angeles CA USA; ^18^ Cardiology I, Department of Cardiology University Medical Center of the Johannes Gutenberg‐University Mainz Mainz Germany

Transcatheter tricuspid valve replacement (TTVR) has emerged as an important treatment tool for patients with severe tricuspid regurgitation (TR) and heart failure.^1^ Currently, one TTVR device is Food and Drug Administration and CE mark approved and several others are at different stages of development. For the transjugular LuX‐Valve Plus TTVR system, evidence is consistently growing. Besides compassionate use 30‐day outcome data,^2^ the TRAVEL study recently reported effective TR reduction (≤2+ in 95.5% of patients) in an Asian patient population, while being associated with low procedural complication rates (permanent pacemaker requirement in 1.6%, severe bleeding in 14.3%) and good 1‐year survival (89.7%).^3^ Since 1‐year real‐world data outside of China are lacking, the present study aimed at investigating 1‐year outcomes following compassionate use LuX‐Valve Plus TTVR.

The study included consecutive patients from 16 international centres who underwent LuX‐Valve Plus TTVR from 2019 until 2024 in a compassionate use programme. Accordingly, no pre‐specified in‐ or exclusion criteria were available and the individual screening process was left to the discretion of each individual centre. Procedural details of TTVR using the LuX‐Valve Plus system have previously been described.^4^ One‐year endpoints included clinical success according to the Tricuspid Valve Academic Research Consortium (TVARC),^5^ TR reduction, 1‐year survival and survival free from heart failure hospitalization (HFH), changes in New York Heart Association (NYHA) functional class and symptoms of right ventricular (RV) failure, RV reverse remodelling (RVRR) as well TTVR‐related adverse events. Numbers are presented as mean ± standard deviation or median with interquartile range (IQR). Two dependent samples were compared using the Wilcoxon test. A two‐sided *p*‐value of <0.05 yielded statistical significance. All analyses were performed using R (version 4.0.4) and SPSS (version 25, IBM, Armonk, NY, USA). The analysis was approved by each centre's local ethics committee and adheres to the principles outlined in the Declaration of Helsinki.

The study included a total of 74 patients (mean age 75.9 ± 7.4 years; 56.8% women). TR was severe, massive and torrential in 13.7%, 21.9% and 64.4%, respectively. Patients were highly symptomatic (NYHA class ≥III 84.3%, peripheral oedema 81.1%, ascites 40.3%, pleural effusion 35.6%) and presented with advanced surgical risk (TRI‐SCORE 5.9 ± 2.4 points). Renal function was impaired as represented by an estimated glomerular filtration rate of 52.4 ± 25.2 ml/min. The median N‐terminal pro‐B‐type natriuretic peptide value was 1985 (IQR 1023–3668) pg/ml. The most common comorbidities within the study cohort were atrial fibrillation (90.4%), arterial hypertension (62.2%) and coronary artery disease (32.9%). TTVR reduced TR to ≤1+ and ≤2+ in 90.5% and 94.6% of patients, respectively. Residual TR was paravalvular in all cases. Intraprocedural success according to TVARC was achieved in 68 patients (91.1%) with a mean procedural time of 131 ± 40 min. The postprocedural tricuspid valve inflow gradient was 2.0 ± 1.2 mmHg. Overall, 42.5% of patients were discharged with prescription of direct oral anticoagulants and 53.5% with vitamin K antagonists. Antiplatelet therapy included acetylsalicylic acid in 5.7% of patients.

One‐year follow‐up was available in 39 out of 41 eligible patients (alive and TTVR performed >1 year ago) (*Figure* *1*). Clinical success at 1‐year follow‐up was 76.9% with durable TR reduction to ≤1+ in 78.4% and ≤2+ in 86.5% of patients (*p* < 0.001). Residual TR was of paravalvular nature in all cases. One‐year survival and survival free from HFH were 77.7% and 66.8%, respectively. At 1‐year follow‐up, NYHA functional class improved to ≤ II in 94.6% of patients (vs. 15.7% at baseline, *p* < 0.001). Beyond that, TTVR was associated with an improvement in heart failure symptoms (peripheral oedema 22.2% vs. 81.1%, ascites 0% vs. 40.3%, pleural effusion 11.1% vs. 35.6%, all *p* < 0.001). While 6‐min walking test distance remained statistically unchanged (baseline 274 ± 119 m; 1‐year follow‐up 295 ± 127 m, *p* = 0.345), the Kansas City Cardiomyopathy Questionnaire (KCCQ) significantly improved (baseline 54.4 ± 23.5; 1‐year follow‐up 68.7 ± 32.5, *p* = 0.017). Echocardiographically, TTVR was associated with numeric RVRR (reduction in RV midventricular diameter from 43.9 ± 8.9 mm at baseline to 36.3 ± 9.7 mm at 1‐year follow‐up, *p* = 0.087) and a decrease in RV function (decrease in tricuspid annular plane systolic excursion from 17.2 ± 4.6 mm at baseline to 15.4 ± 5.5 mm at 1‐year follow‐up, *p* = 0.024; RV fractional area change from 37.3 ± 9.0% to 35.6 ± 8.0%, *p* = 0.249). Within the whole study period no events of myocardial infarction, stroke, pulmonary embolism, or valve thrombosis were noted. While five patients (6.7%) suffered bleeding complications within the index hospitalization, no further bleeding events were noted during follow‐up. No association between bleeding complications and antiplatelet or anticoagulation therapy was observed. Among 50 pacemaker naïve individuals, three patients (6.0%) required permanent pacemaker implantation within the first 30 days after the procedure with one further patient 8 months after TTVR due to atrial fibrillation with slow ventricular response (cumulative incidence at 1 year 8.0%).

**Figure 1:**
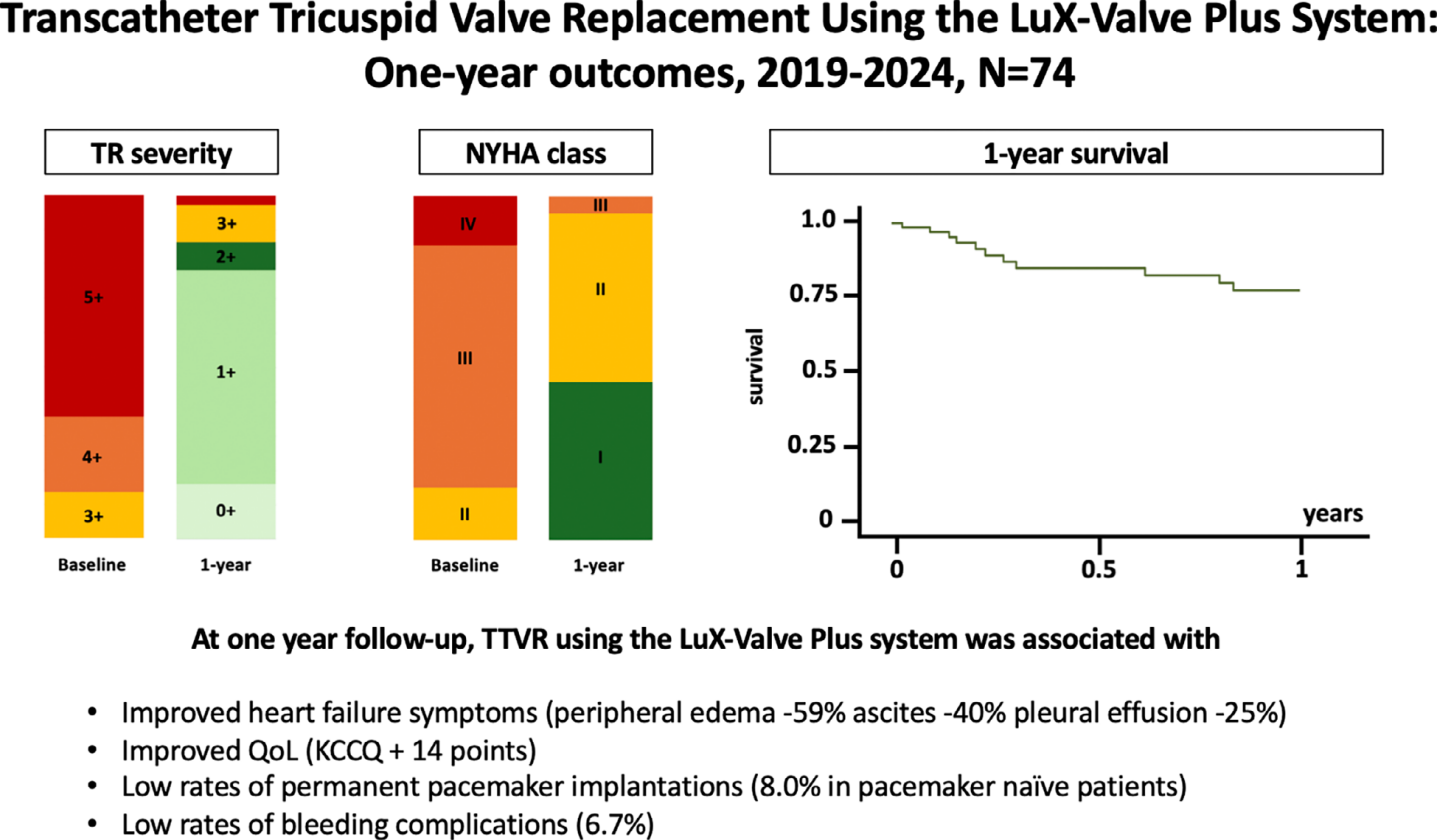
At 1‐year follow‐up, transcatheter tricuspid valve replacement (TTVR) using the LuX‐Valve Plus system is associated with durable tricuspid regurgitation (TR) reduction, low rates of bleeding complications and permanent pacemaker implantation as well as an improvement in quality of life and heart failure symptoms. KCCQ, Kansas City Cardiomyopathy Questionnaire; NYHA, New York Heart Association; QoL, quality of life.

In summary, this is the largest available compassionate use LuX‐Valve Plus TTVR experience available today. Compared to controlled data from the recently published TRAVEL trial, 1‐year survival and survival free from HFH were lower among real‐world patients (78% vs. 90% and 67% vs. 85% for this study vs. TRAVEL, respectively) probably reflecting a more selected patient cohort within the trial environment.^3^ Beyond that, many patients within this compassionate use cohort were ineligible for alternative treatment options due to comorbidities or challenging anatomical conditions. Comparable results were observed for TR reduction to ≤2+ (87% vs. 95%). With a cumulative pacemaker implantation rate of 8.0% of pacemaker naïve patients at 1 year, numbers were higher under real‐world conditions compared to trial data (1.6%). Noteworthy, those numbers are lower compared to other TTVR devices with more radial anchoring force (e.g. ~25% of pacemaker naïve patients in TRISCEND II).^6^ Alike reported in the TRAVEL study, TTVR using the LuX‐Valve Plus system was associated with an improvement in quality of life as assessed by the KCCQ. In line with previous reports, RV function numerically declined after TTVR reflecting a pseudonormalization of RV function from a previously hyperactive state.^7^, ^8^, ^9^ This study is subject to a number of limitations, primarily due to the retrospective nature of the registry (no control group, no echocardiographic core laboratory or clinical event adjudication committee). However, echocardiographic analyses and reporting of clinical events were performed by experienced physicians at each study centre.

In conclusion, compassionate use LuX‐Valve Plus TTVR was associated with durable TR reduction over the course of 1‐year follow‐up with an observed improvement in heart failure and TR symptoms as well as RVRR. As previously reported for TTVR, the most common adverse events included need for permanent pacemaker implantation and bleeding complications.

**Conflict of interest**: L.S. received speaker honoraria from Edwards Lifesciences. O.d.B. received institutional research grants and consulting fees from Abbott, Boston Scientific, Medtronic and SMT. J.E.M. received institutional research grant from Johnson and Jonson Heart Recovery, Novo Nordisk Foundation, speaker fee from Johnson and Jonson Heart Recovery and Abbott, advisory board from Boston Scientific and Magenta. K.C.Y.S. is physician proctor for Abbott Structural Heart, Boston Scientific, Edwards and Medtronic; and serves as consultant for Venus Medtech and Jenscare. A.C. is proctor for Abbott Vascular and received speaker fees for Abbott Vascular, Edwards Lifescience, GE Healthcare, BMS, MSD, Pfizer. J.D. received consulting fees from Abbott and Edwards Lifesciences. M.N. received proctoring and consulting fees from Abbott Vascular, Medtronic, Robocath. R.E.L. received speaker fees from Abbott, Edwards, Boston, Venus Medtech, Jenscare and Valgen. K.P.K. is a consultant to Edwards Lifesciences and ReCor Medical. J.H. received research support and speaker honoraria from Edwards Lifesciences. All other authors have nothing to disclose.

## Appendix A PubMed‐indexed investigator list

Ralph Stephan von Bardeleben^18^, Sebastian Rosch^18^, André Vincentelli^9^, Gilles Lemesle^9^.
